# Editorial: Cerebral Small Vessel Diseases: From Vessel Alterations to Cortical Parenchymal Injury

**DOI:** 10.3389/fneur.2020.00092

**Published:** 2020-02-14

**Authors:** Susanne J. van Veluw, Eric Jouvent, Andreas Charidimou

**Affiliations:** ^1^Department of Neurology, Massachusetts General Hospital and Harvard Medical School, Boston, MA, United States; ^2^Department of Neurology, APHP, Lariboisière Hospital, Paris, France; ^3^Univ Paris Diderot, Sorbonne Paris Cité, Paris, France; ^4^UMR-S 1141 INSERM, Paris, France; ^5^Boston Medical Center, Boston University, Boston, MA, United States

**Keywords:** small vessel disease, cerebral amyloid angiopathy, perivascular spaces, MRI, animal models

Cerebral small vessel disease (CSVD) encompasses a group of (mostly) age-related and vascular risk factor-driven processes affecting the small vessels of the brain. The two most common forms of CSVD are sporadic hypertension-related microangiopathies and cerebral amyloid angiopathy (CAA) (Aghetti et al.; Yilmaz et al.), which can both result in ischemic or hemorrhagic damage including clinically overt strokes (Blanc et al.; Chen Z. et al.; Wang et al.). During life, CSVD is recognized by its manifestations on magnetic resonance imaging (MRI) scans, including white matter hyperintensities, lacunes, cerebral microbleeds, microinfarcts, cortical superficial siderosis, and MRI-visible perivascular spaces (Yilmaz et al.; Appleton et al.; Chen Z. et al.; Catak et al.; Ferro et al.; Frey et al.; Gyanwali et al.; Muñoz Maniega et al.; Schirmer et al.). These manifestations of CSVD might collectively contribute to cognitive decline in older individuals (Huo et al.; Liu et al.; Yilmaz et al.). Currently, there are no specific therapies or prevention strategies that can slow down the progression of cognitive impairment in these patients. Some of the major existing challenges in the field, that have hampered the identification and development of therapeutic targets are the following: (1) conventional MRI does not capture the entire spectrum of CSVD manifestations nor does it directly show the smallest blood vessels in the brain, (2) the histopathological features underlying some of the CSVD manifestations on MRI are not fully understood, (3) it is unclear how changes to the small vessels result in parenchymal tissue injury either locally or in remote (including cortical) structures, and (4) it is poorly understood how CSVD manifestations contribute to clinical and cognitive deficits in older individuals (and appears context dependent). Recent advancements in neuroimaging techniques have fueled scientific research into these knowledge gaps. The aim of this topic was to bring together novel studies in this area, and to highlight different yet complementary approaches and techniques to advance our understanding of CSVD.

The large number of contributions made to this special issue underscores the growing interest in CSVD research, and at the same time the complexity of these processes. The studies covered a wide range of topics, between novel image analysis approaches and the identification of new neuroimaging signatures with relevance to CSVD (Chen Y. C. et al.; De Guio et al.; Frey et al.; Liu et al.; Muñoz Maniega et al.; Peng et al.), to the uncovering of etiologies and potential mechanisms underlying tissue injury (Blanc et al.; De Guio et al.; Ferro et al.; Uemura et al.; Zhu et al.). Notably, several contributions zoomed in on MRI-visible perivascular spaces, the spaces surrounding long penetrating arterioles of the brain (Gyanwali et al.; Huo et al.; Wang et al.; Yilmaz et al.). Perivascular spaces are believed to be related to clearance of waste products from the brain, and when enlarged, may implicate impaired perivascular drainage as a potential mechanism. In particular, impaired perivascular clearance of Amyloid β from the cortex has been implicated in the pathophysiology of Alzheimer's disease and dementia. This pathway may therefore represent an interesting target to direct the development of therapeutic or prevention strategies. In this scenario, MRI-visible perivascular spaces may be a useful biomarker in future clinical trials. MRI-visible perivascular spaces in the basal ganglia have been linked to hypertension, whereas in the cerebral white matter (centrum semiovale) the presence of perivascular spaces suggests advanced CAA in the overlying cortex. Whether both patterns are related to faulty waste clearance from subcortical or cortical parts of the brain respectively, is currently not completely clear, and requires further experimental investigations (Wang et al.)

Despite some of the latest advancements in CSVD research, there is still a great unmet need for the development of better disease models for experimental studies to unravel the pathophysiological mechanisms involved in CSVD (Michael et al.) Due to the scarcity of relevant animal models to recapitulate several of the aspects of small vessel injury and the formation of spontaneous lesions, we have an imperfect understanding of the disease pathways. Future studies are warranted to take on this challenge with the hope to bridge the gap from the bench to the bed side and improve clinical outcome in patients affected with CSVD.

## Author Contributions

SV drafted the editorial. EJ and AC made substantial revisions.

### Conflict of Interest

The authors declare that the research was conducted in the absence of any commercial or financial relationships that could be construed as a potential conflict of interest.

